# Nanostructured Synthetic Carbons Obtained by Pyrolysis of Spherical Acrylonitrile/Divinylbenzene Copolymers

**DOI:** 10.1371/journal.pone.0043354

**Published:** 2012-08-16

**Authors:** Danish J. Malik, Andrzej W. Trochimczuk, Sylwia Ronka

**Affiliations:** 1 Department of Chemical Engineering, University of Loughborough, Loughborough, United Kingdom; 2 Faculty of Chemistry, Wroclaw University of Technology, Wroclaw, Poland; University of Akron, United States of America

## Abstract

Novel carbon materials have been prepared by the carbonization of acrylonitrile (AN)/divinylbenzene (DVB) suspension porous copolymers having nominal crosslinking degrees in the range of 30–70% and obtained in the presence of various amounts of porogens. The carbons were obtained by pre-oxidation of AN/DVB copolymers at 250−350°C in air followed by pyrolysis at 850°C in an N_2_ atmosphere. Both processes were carried out in one furnace and the resulting material needed no further activation. Resulting materials were characterized by XPS and low temperature nitrogen adsorption/desorption. It was found that maximum pyrolysis yield was ca. 50% depending on the oxidation conditions but almost independent of the crosslinking degree of the polymers. Porous structure of the carbons was characterized for the presence of micropores and macropores, when obtained from highly crosslinked polymers or polymers oxidized at 350°C and meso- and macropores in all other cases. The latter pores are prevailing in the structure of carbons obtained from less porous AN/DVB resins. Specific surface area (BET) of polymer derived carbons can vary between 440 m^2^/g and 250 m^2^/g depending on the amount of porogen used in the synthesis of the AN/DVB polymeric precursors.

## Introduction

Polymer-derived carbons are attracting increasing interest in the scientific and technological communities because such materials are finding widespread applications in the field of catalysis, gas storage, in many separations, including environmental and biomedical ones. They display considerable adsorption capacity due to high specific surface area and are more stable and mechanically robust than other types of carbonaceous materials. Many research teams have determined the effect of various parameters governing the structure and properties of polymer derived carbons. These parameters include type of polymer, activation conditions, pyrolysis temperature and pyrolysis atmosphere.

A review of the scientific literature on polymer pyrolysed carbons highlighted earlier work on carbonization of polyacrylonitrile [1], [2] which has been very widely studied with respect to the formation of carbon fibers [3]–[5]. Other published studies on polymer derived carbons include polyimides [6], [7], various phenol-formaldehyde resins [8], [9], polyvinylpyridine and poly(N-vinylcarbazole) [10]. Additionally, polymers in the shape of beads have received some attention due to the good hydrodynamic properties of the resultant carbons in fixed-bed/fluidized-bed type separation applications. Early work on carbonization of spherical, suspension polymers was carried out by Neely [11] and continued by numerous research groups in the ‘80 s and ’90 s [12]–[15]. Latest examples include glycidyl methacrylate-co-ethylene glycol dimethacrylate, bead cellulose and poly(acrylonitrile-co-divinylbenzene) [16], 4,4′-bis(maleimidodiphenyl)methane and divinylbenzene copolymers [17], sulfonated poly(styrene-co-divinylbenzene) [18], phosphorylated poly(styrene-co-divinylbenzene) [19], sulfonated divinylbiphenyl copolymers [20], phosphoric acid activated chloromethylated and sulfonated poly(styrene-co-divinylbenzene) [21], [22].

In contrast to activated carbons obtained conventionally from wood, coal and other natural products, the pore structure of polymer-derived carbons can be better controlled by the choice of the precursor material i.e. its own porosity, chemical composition and pore distribution plus choice of carbonization and activation conditions.

Our interest in the carbonization of polymeric resins originated from our research in haemoperfusion in which we realized the need for new sorptive materials having heteroatoms in their structure. These heteroatoms (e.g. nitrogen) provide desired changes to the sorptive properties of the polymer-derived carbon surface and also change the surface polarity and biocompatibility [23]. Also, it would be interesting to determine if the original porosity of the polymeric precursor is preserved in the final material. The aim is to be able to prepare tunable nanostructured carbon materials with desired pore structure which would be immensely valuable for the development of effective adsorbents for large solutes, e.g. some glycosylated proteins and cytokines typically encountered in the treatment of patients with renal failure and sepsis.

The aim of the current work was to investigate how the pore structure and chemical composition of synthesized acrylonitrile/divinylbenzene porous copolymers influence the yield, pore structure and composition of resulting carbons derived from these polymers.

**Figure 1:**
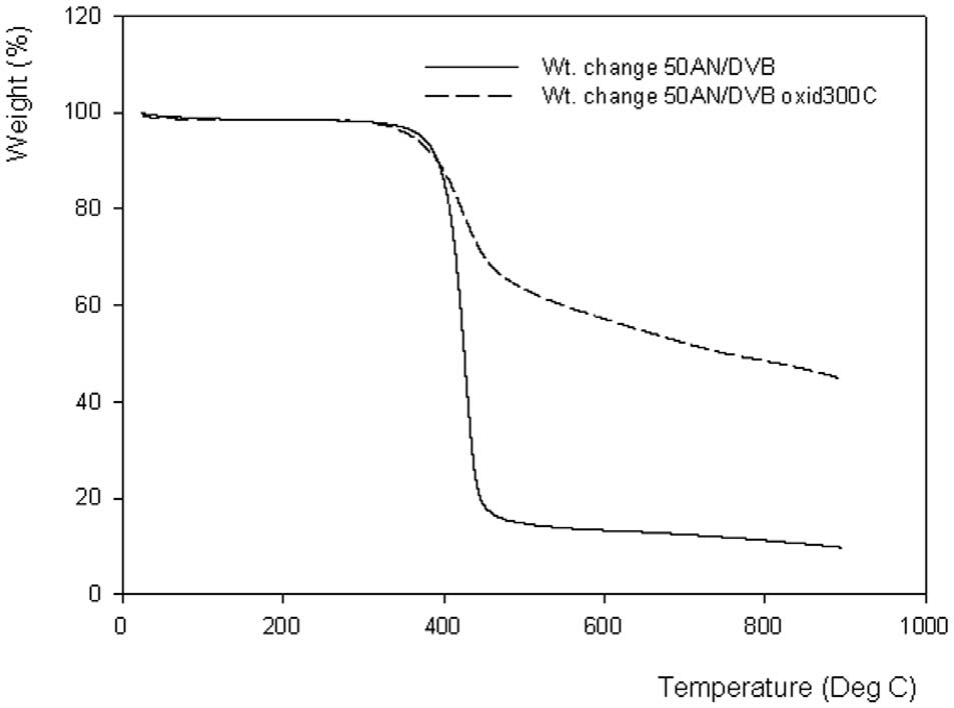
Thermogravimetric curves for AN/DVB and oxidized AN/DVB samples.

**Table1:** Oxidation of sample 30AN/DVB 0.33 at different temperatures.

Temperature, °C	Yield, %	Nitrogen content, mmol/g %
250	96	9.16 12.8
300	82	7.95 11.1
350	64	8.66 12.1

Nitrogen content of starting 30AN/DVB 0.33 polymer −9.84 mmol/g (13.8%).

**Table 2:** Polymer-derived carbons characteristics.

Carbon symbol	Nitrogen content mmol/g	Yield, %	NH_3_ released mmol/g	HCN released mmol/g	Specific surface area m^2^/g	Pore volume cm^3^/g
30AN 0.50 ox250C	4.7	35	5.8	1.2	187	0.39
30AN 0.50 ox300C	4.7	46	5.7	1.2	429	0.59
30AN 0.50 ox350C	4.8	41	4.9	1.2	439	0.60
30AN 0.33 ox250C	4.0	36	5.3	1.9	115	0.20
30AN 0.33 ox300C	4.0	50	5.5	1.6	212	0.25
30AN 0.33 ox350C	4.5	42	5.3	3.3	246	0.23

## Materials and Methods

### Synthesis of Polymers and their Modification

Acrylonitrile/divinylbenzene copolymers were prepared in the form of spherical beads using the process of suspension polymerization. Polymers were prepared in the presence of inert diluents in order to make them porous. A typical preparation procedure involves suspending the mixture of monomers (acrylonitrile and divinylbenzene), an appropriate amount of inert diluents (nonane and toluene 1∶9 w/w) and the initiator benzoyl peroxide (0.5 wt. % with respect to amount of monomers used) in a three necked glass reactor filled with aqueous phase. The aqueous phase consists of 5 wt.% calcium chloride and a suspension stabilizer Gohsenol GH-23 (1 wt.% with respect to the amount of organic phase). Full details of the polymerization are given in [24]. Nitrogen elemental analysis was done on samples mineralized using concentrated sulfuric acid followed by the Kjeldahl method. Water regain of the resins was measured using the centrifugation method and calculated as:
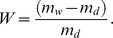
where m_w_ is the weight of polymer after centrifugation in a small column with fritted-glass bottom and m_d_ is the weight of polymer after drying at 100°C overnight.

**Figure 2:**
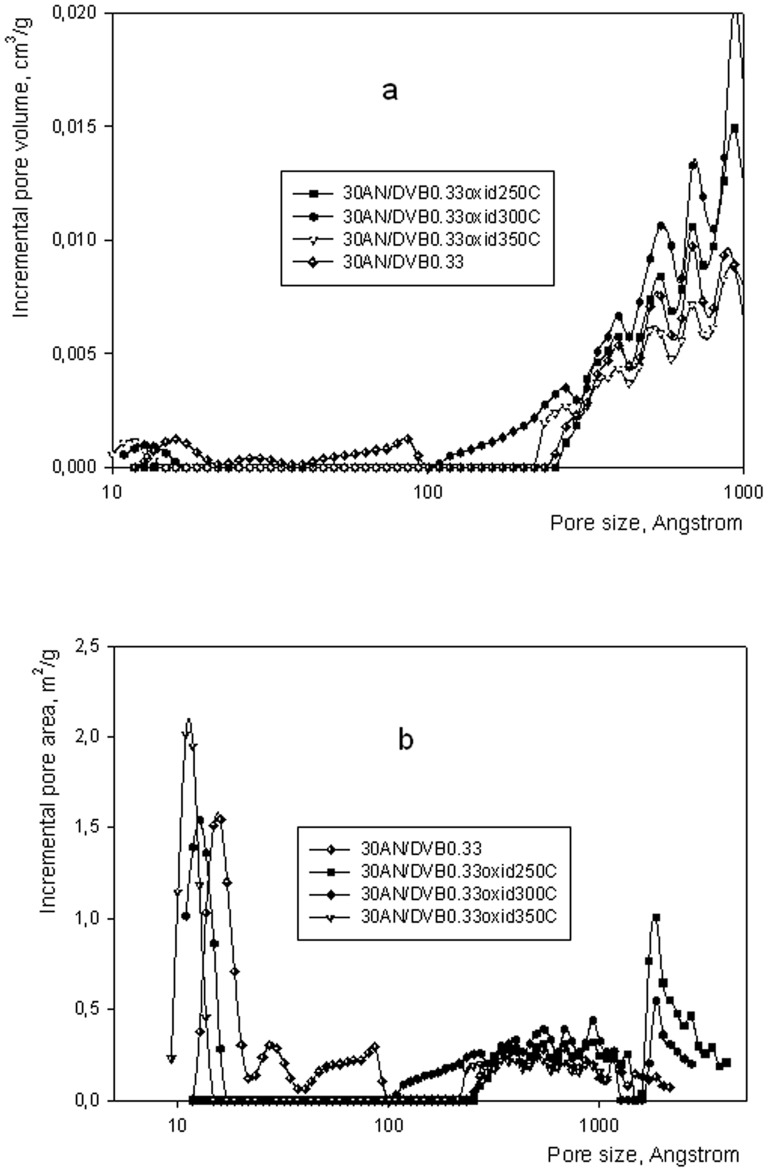
Incremental pore volume (a) and incremental pore area (b) of oxidized 30AN/DVB 0.33 polymers.

**Figure 3:**
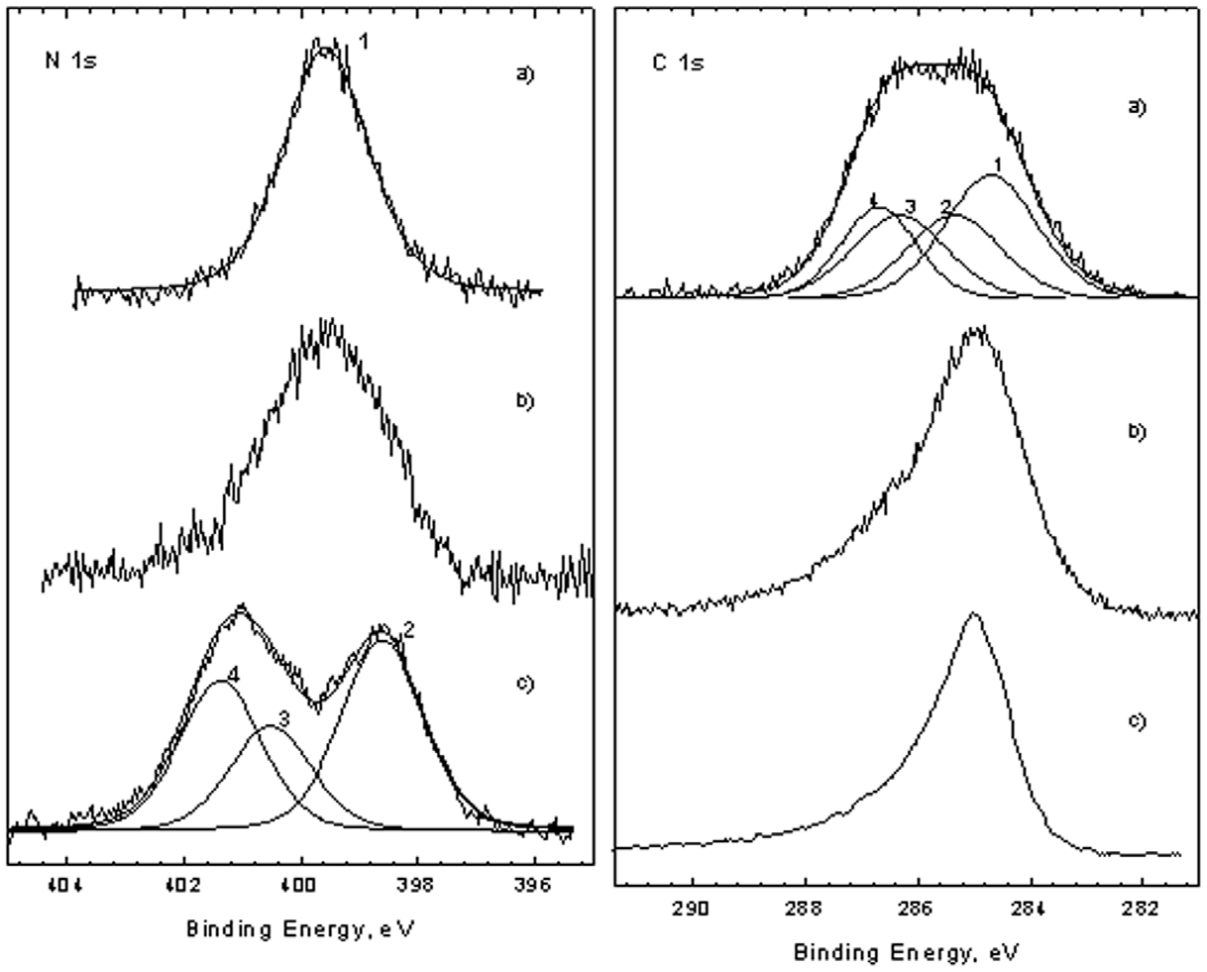
Nitrogen (A) and carbon (B) XPS spectra of AN/DVB copolymer (a), oxidized sample (b) and carbon (c).

**Figure 4:**
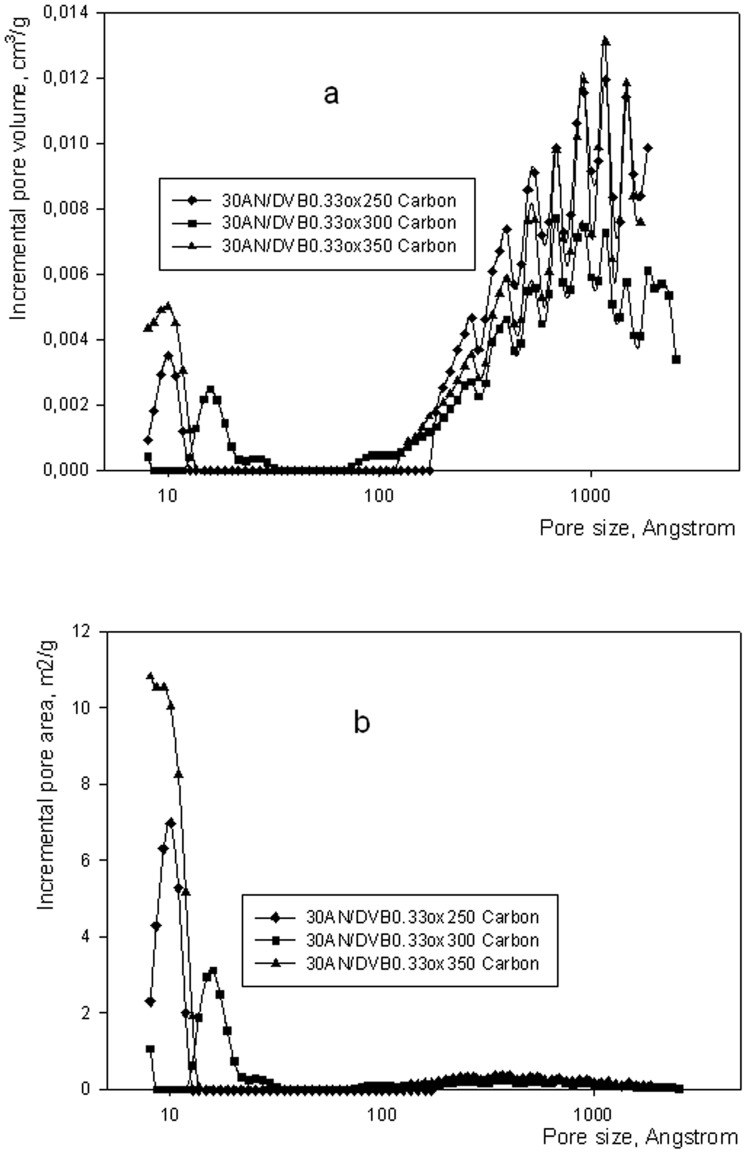
Incremental pore volume (a) and incremental pore area (b) of carbons obtained from 30AN/DVB 0.33 oxidized polymers.

**Figure 5:**
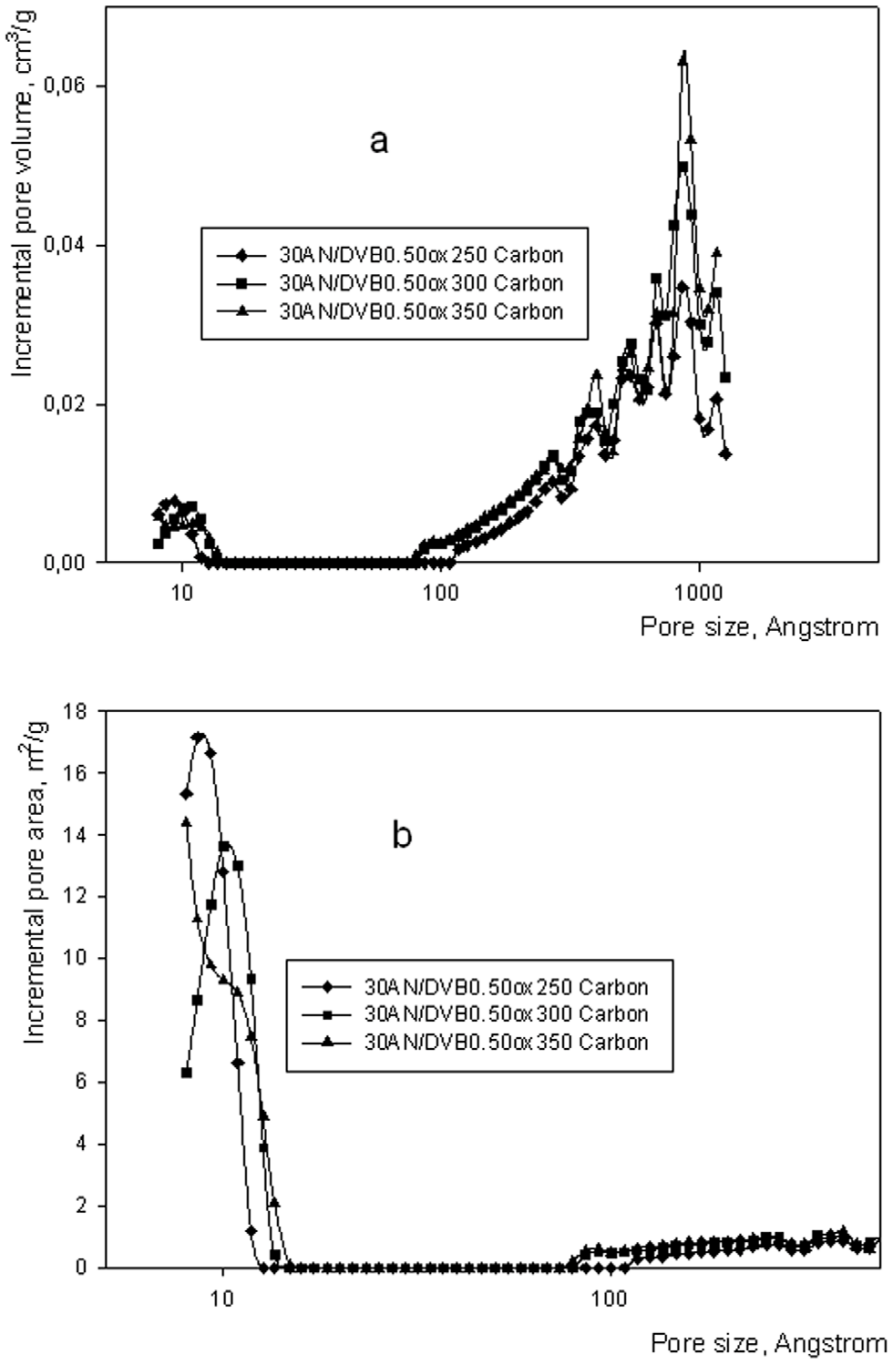
Incremental pore volume (a) and incremental pore area (b) of carbons obtained from 30AN/DVB 0.50 oxidized polymers.

### Pyrolysis of Polymer Samples

**Figure 6:**
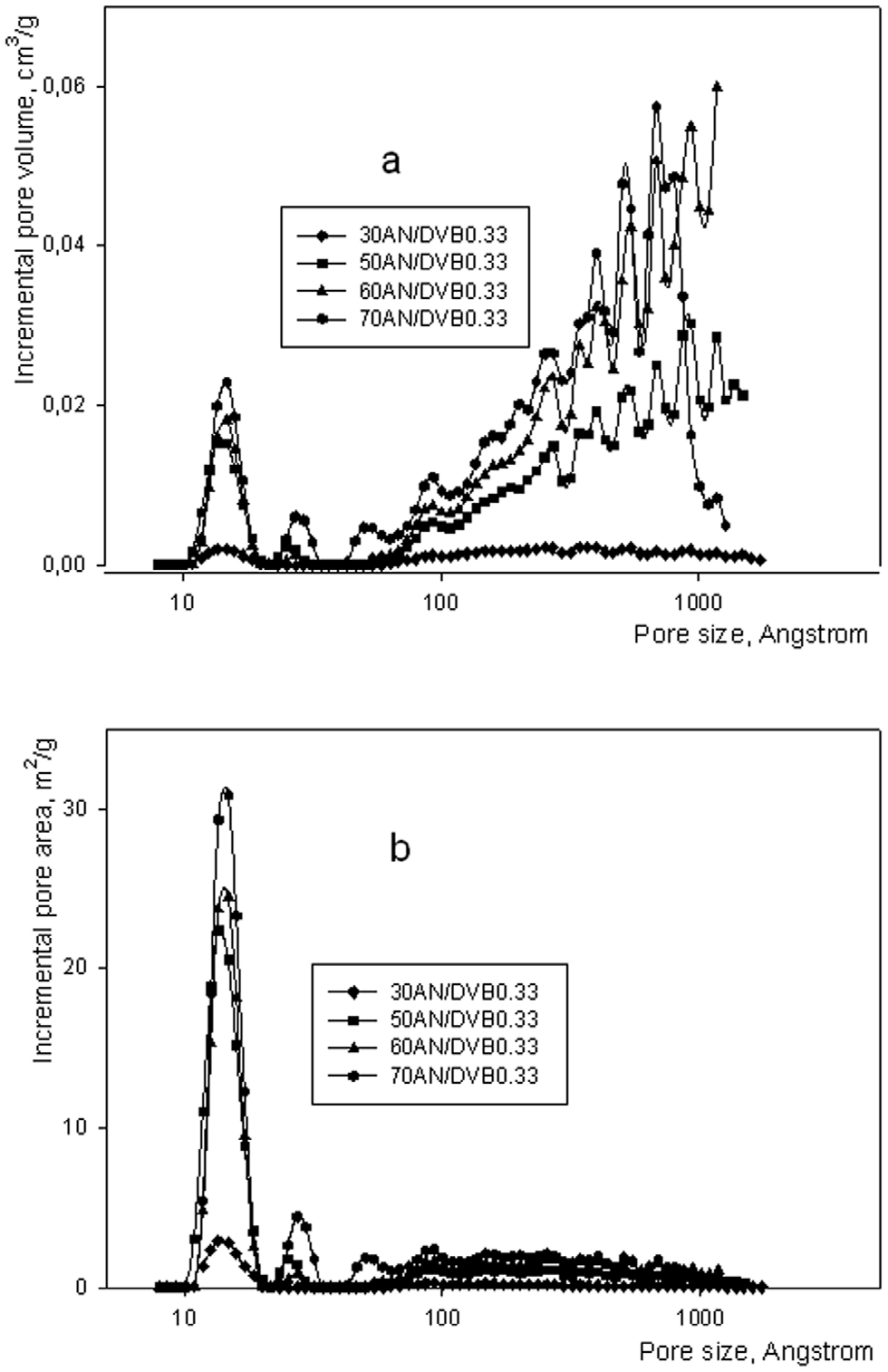
Incremental pore volume (a) and incremental pore area (b) of AN/DVB 0.33 polymers having various crosslinking degree.

**Table 3:** Yield and nitrogen content of carbons as a function of crosslinking degree and amount of nitrile groups in polymeric precursor.

Polymer crosslinkingdegree, wt.%	Yield of carbon,%	Nitrogen content,mmol/g		Surface area,m^2^/g		Pore volume,cm^3^/g	
		polym. carb.		polym. carb.		polym. carb.	
30	50	9.84	4.70	34	212	0.15	0.25
50	51	5.94	2.03	460	59	0.96	0.30
60	46	3.61	1.52	560	162	1.33	0.57
70	48	1.90	1.17	720	343	1.54	0.53

all polymers are of 0.33 series, oxidized at 300°C, carbonized at 850°C.

**Figure 7:**
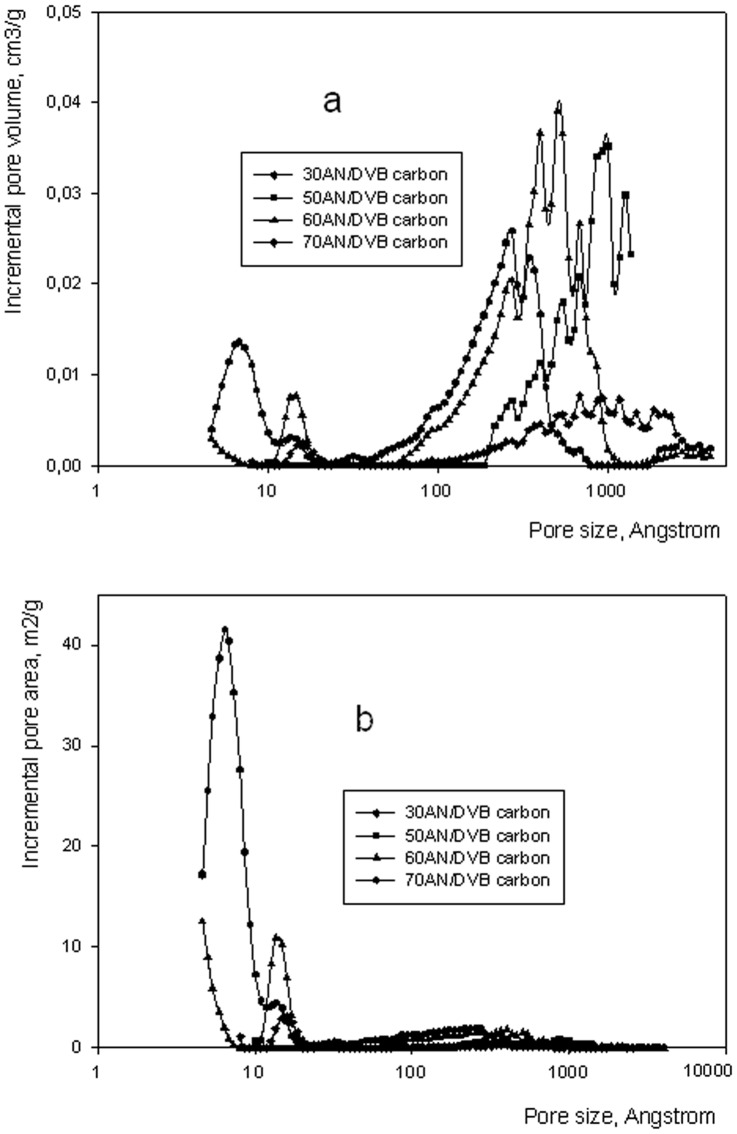
Incremental pore volume (a) and incremental pore area (b) of carbons obtained from AN/DVB 0.33 polymers of various crosslinking degree.

Pyrolysis of polymer samples involved heating small quantities (typically ∼1 g) of dry polymer placed in a shallow quartz crucible in a horizontal tube furnace. Experiments were carried out in two stages. Firstly, the AN/DVN polymer samples were oxidized by heating under an air atmosphere (air flow rate 100 mL/min) at a specific temperature (250–350°C). The temperature was raised from ambient to the final set temperature at a rate of 50°C /hr and oxidizing soak time at the final temperature was 1 hr. Secondly, the sample was flushed with nitrogen for 1 hr at the final oxidizing temperature (N_2_ flow set at 400 mL/min). The oxidized polymer sample was subsequently thermally treated at 400°C for 4 hr and then the temperature was raised at a rate of 100°C /hr to a final temperature of 850°C with a final soak time of 1 hr at this temperature. The sample was then allowed to cool to room temperature in a nitrogen atmosphere. The polymer-derived carbon was stored in a desiccators for subsequent characterization analysis.

Samples of polymers used in this work were designated on the basis of the nominal crosslinking degree followed by AN/DVB and the ratio of monomers to the sum of monomers and inert diluents used during polymerization. So, for example 30AN/DVB 0.33 refers to a sample of AN/DVB polymer containing 30 wt.% of crosslinker and obtained in the presence of two volumes of inert diluents with respect to monomers. Oxidized and carbonized samples were designated by addition of the oxidizing temperature and the letter C to the description of the sample, respectively.

### Physical Characterization of Samples

Specific surface area and pore size distribution measurements were carried out using low temperature (77 K) nitrogen adsorption-desorption isotherms (Micromeritics ASAP 2010). A high sensitivity 1 Torr pressure transducer was used to ensure high precision of readings at the low pressure values. Samples of polymer-derived carbons were degassed under vacuum at 110°C for 24 h prior to measurement of the adsorption-desorption isotherms.

XPS experiments were carried out using a SPECS UHV System equipped with a PHOIBOS 100 spectrometer and SPECLAB software. X-rays were generated using a magnesium anode operating at 100 W (wide angle scan) and 200–300 W (high resolution spectra). The spectrometer energy scale was calibrated using Au (4f7/2), Ag (3d5/2) and Cu (2p3/2) lines at 84,2, 367,9 and 932,4 eV, respectively. The analyzer mode was set at a constant serving of energy of 30 eV (survey scan) and 5 eV (narrow scan). Sample charging was compensated using an electrode flood gun with 0.5 mA current and 0.1 eV energy. The detection angle was normal near the surface. The base pressure in the UHV analysis was below 1×10^−9^ mbar. The peaks were fitted and derived by SPECSLAB and Origin 7.0 professional software packages using Gaussian-Lorenzian curve profile and Shirley baseline correction.

Thermogravimetric (TG) analysis was performed using a TA Instruments model 2950, fitted with a silica-lined EGA furnace for better atmosphere control. Samples of ca. 10 mg of the resin were contained in 10 mm diameter shallow platinum pans, so as to form a bed-layer of a single particle diameter in depth for improved heat and mass transfer. The materials were heated at 3°C/min under an oxygen-free nitrogen atmosphere (gas flow set at 100 mL/min).

## Results and Discussion

It is widely accepted that oxidation of polyacrylonitrile is a crucial step in order to successfully carbonize this material [25]. The oxidation starts-off the cyclization reaction between the nitrogen of one nitrile group and the carbon of a neighbouring nitrile group; this requires the presence of a nucleophile stronger than the nitrile nitrogen. Oxidation performed either with wet oxidizing agents such as hydrogen peroxide or with air at elevated temperatures causes oxidation of some of the nitrile groups to carboxylic and other oxygen containing ones (e.g. carbonyl, hydroxyl and lactone). Carboxylic groups can in turn initiate cyclization of acrylonitrile sequences of mers leading to pyridinic type structures. The effect of the oxidation on the yield of the material obtained by AN/DVB carbonization is illustrated in Figure 1. Despite the presence of a crosslinker, the carbonization yield of the 50AN/DVB sample is only ca. 10%, whereas the same polymer oxidized at 300°C gave a yield of ca. 45%. Air oxidation at three different oxidation temperatures were chosen in this study with the objective of achieving the highest possible carbon yield. The results of oxidation of 30AN/DVB 0.33 polymers are presented in Table 1 and the porous structure of the oxidized samples is illustrated in Figure 2a. Of the three oxidation temperatures, only the oxidation at 250°C does not result in a significant weight change in the resulting oxidized polymer (see Table 1) and its nitrogen content. Increasing the oxidation temperature to 300°C and 350°C results in a decrease of weight to 82 and 64% of the starting polymer weight. A relative increase of nitrogen content in the material oxidized at 350°C is indicative of the higher carbon loss rather than nitrogen loss from the polymeric backbone. This weight loss at temperatures in the range 250–350°C can be ascribed to dehydrogenation and to loss of nitrogen due to formation of ammonia and hydrogen cyanide. Table 2 shows higher amounts of ammonia being evolved from the 30AN/DVB 0.50 polymer at lower oxidation temperature, whereas HCN is evolved at the same level at all three temperatures. However, oxidation of 30AN/DVB 0.33 polymer leads to higher amounts of hydrogen cyanide being evolved at all temperatures and consequently to a lower nitrogen content in the oxidized samples. Since the 30 AN/DVB 0.33 sample is less porous than 30 AN/DVB 0.50 it may be hypothesized that oxygen transport within the material is slower in the former polymer. This case illustrates the importance of the physical structure of the polymers in the oxidation and carbonization processes.

Two types of polymers were subjected to oxidation and subsequent carbonization. One consisted of AN/DVB polymer, which had been obtained in the presence of a smaller proportion of inert diluents −0.5 series, whereas the other was synthesized in the presence of a higher amount of the same inert diluents. The amount of diluents influences the porous structure in the AN/DVB copolymers and this feature is passed on to carbons derived from them. The porous structure of the oxidized samples is shown in Figures 2a and 2b. Treatment of 30 AN/DVB 0.33 polymer with air at elevated temperatures results in moderate changes in its porous structure. Incremental pore volume shows a slight shift of peaks corresponding to small pores from ca. 30 angstroms to ca. 12–20 angstroms for samples oxidized at 300 and 350°C. The volume of macropores in the samples also appears to increase compared to the original polymer (see Figure 2a). A shift in the micropores can be seen in Figure 2b, where there is a clear indication of the presence of smaller pores in samples oxidized at higher temperatures.

Figure 3 shows XPS spectra of 30AN/DVB 0.33 copolymer, the sample after oxidation and the resultant carbon. The N_1s_ peak (see N_1s_ (trace a) in Figure 3) at binding energy 399.57 eV is symmetrical and entirely due to the nitrogen in nitrile groups of 30AN/DVB 0.33 sample. After oxidation the position of the maximum of N_1s_ peak does not change. This means that nitrogen is still mostly in the nitrile groups. However, the peak width at half maximum (FWHM) increases substantially from 1.75 to 2.42 eV (compared to the starting copolymer with the same pass energy (5eV)). Slight asymmetry of the peak and the presence of a shoulder shows that some additional bonds are appearing. Most likely, this could be nitrogen present in the amides of acrylic acid, as they are the first product of oxidation of acrylonitrile mers. Deconvolution of N_1s_ after carbonization (see Figure 3, trace c) assumes peaks attributed to pyridinic type N-6 (398.6eV), pyrrolic type N-5 (400.5eV) and nitrogen in condensed aromatic systems type N-Q (401.3eV). Such peaks were identified in the work of Kapteijn at al [26] as the most prevailing in the polyacrylonitrile char after carbonization at 1073 K. The choice of the number and type (position) of peaks in a deconvolution procedure is arbitrary but in the present case this is supported by the fact that in Kapteijn at al [26] polyacrylonitrile was carbonized at 1073K and in the present case at 1123 K. Application of this deconvolution gave relative proportions of 43% (atom %), 23.5% and 33.5% of peaks attributed to N-6, N-5 and N-Q. These results are close to those observed in [26], however nitrogen in condensed aromatic system type bonds are more visible in the present case. This observation can be explained by the presence of aromatic rings originating in the divinylbenzene crosslinker in the AN/DVB resins. Such aromatic structures can condense with pyridinic structures (N-6) obtained during carbonization, thus giving N-Q structures. Deconvolution was done assuming FWHM 1.66eV for all peaks and assuming identical peak shapes (Lorentzian/Gaussian  = 0.7).

All carbon XPS spectra were calibrated for N_1s_ at 399.57 eV. C_1s_ spectrum of the starting 30AN/DVB 0.33 copolymer was deconvoluted and this resulted in peaks for four separate bonds: CH (aromatic) at 284.7eV, -CH_2_- at 285.35eV, -CH(CN) at 286.33eV and -CN at 286.7eV. Calculated areas under each peak were in good agreement with the stoichiometric amount of each of the above bonds in the starting polymer. The C_1s_ peak undergoes bigger changes upon oxidation (trace b) and the maximum of the peak arose at 285 eV which can be explained by coverage of the surface of the sample by carbon from the atmosphere. This is additionally supported by the increase of the C:N ratio from 6∶1 to 8∶1 after oxidation. After carbonisation (trace c) FWHM decreases compared to the oxidised polymer from 2.2 eV to 1.7 eV. This is typical for more homogeneous materials, however, carbon adsorbed from the air appears dominating at the surface. But, a peak at 284.65 eV characteristic for C-aromatic is increasingly visible.

Porous structure of carbons obtained by oxidizing 30AN/DVB 0.33 polymers oxidized at different temperatures is presented in Figure 4a and 4b. 30AN/DVB 0.33 oxidized at 350°C and carbonized at 850°C displays well-developed microporosity and the highest amount of macropores in comparison with all other carbons in this series. Carbonization of samples 30AN/DVB 0.50 follows exactly the same pattern as for sample 30AN/DVB 0.33 (see Figure 5a and 5b). Since the yield of the carbon and the developed porous structure of samples oxidized at 300°C seem the optimal ones, it was decided that this oxidizing temperature would be applied in further experiments leading to polymer derived-carbons from polymers of higher degree of crosslinking.

Details of the porous structure of AN/DVB polymers and of AN/DVB derived carbons are shown in Figure 6a and 6b and Figure 7a and 7b respectively. As can be seen in Figure 6a and 6b and Table 3, pore volume and area increased by increasing the DVB content in AN/DVB polymers. Highest values are displayed by the most crosslinked polymer. The polymer with the lowest crosslinking level contains very small amounts of micropores. Upon oxidation and carbonization all samples, with the exception of 30AN/DVB, display a decrease in the pore volume and specific surface area (Table 3, Figure7a and 7b). This decrease is seen in both the micropore and macropore range and is very substantial for samples crosslinked with 50–70% of DVB. The decrease in pore volume and specific surface area of highly crosslinked samples is accompanied by a polymer weight loss is in the vicinity of 50%. It can be assumed that the porous structure typical for highly crosslinked polymers obtained in the presence of a large proportion of inert diluents and characterized by large pore volumes in the samples collapses during the thermal treatment. Of some interest is that the weight loss is almost independent of the amount of nitrile groups in the starting polymers. This result is rather unexpected since thermal stability and high carbon yield is usually ascribed to the cyclization of acrylonitrile mers and dehydrogenation leading to the formation of pyridinic type structures in the carbonized material [25]. These structures can condense at temperatures above 700°C giving nitrogen and multimember aromatic ring structures. However, in this work, it is seen that a larger proportion of nitrogen is retained in carbon materials (with respect to the original nitrogen content in the starting samples) prepared from polymers 60 and 70AN/DVB 0.33. In the latter sample 1.17 mmol of nitrogen/g was found in the resulting carbon, corresponding to 62% of the original value. This may suggest that at low overall nitrogen concentration, the condensation reactions mentioned above are not feasible since probability of finding two or more pyridinic type rings next to each other is greatly reduced. Thus, suppression of this reaction may lead to retention of nitrogen in carbons prepared from highly crosslinked polymers. The constant yield of carbons obtained from AN/DVB polymers with greatly reduced nitrogen content might be explained in terms of an oxidation step. The oxidation reaction may bring into play aromaticity of these polymers i.e. the DVB rich part of the polymers. For example, unreacted vinyl bonds can be relatively easily oxidized. Stabilizing effect of air oxidation of DVB polymers has been recorded in the literature [27]. However, because of large differences in the experimental conditions between the cited work and here no definitive conclusion can be drawn. Problems concerning oxidative stabilization of the AN/DVB polymers will form the scope of a separate work which will be published in due course.

Comparison of the carbonization behavior of suspension acrylonitrile/divinylbenzene copolymers shows that the initial distribution of pores is largely preserved in the resulting carbons although total pore volume and surface area is decreased. Additionally, a subset of micropores appears in carbon samples obtained from highly crosslinked polymers. Yield of carbonization is almost independent of the crosslinking degree and of the nitrile group content in the starting polymers. This allows preparation of polymer derived carbons with various amounts of nitrogen heteroatoms in the carbon matrix and potentially variable surface characteristics of the resulting carbons. The carbons prepared using the methods employed here need no activation, which simplifies the synthesis process. The sorptive properties of these carbon materials have recently been published separately [28].
